# Thawing Rate Predicts Acute Pulmonary Vein Isolation after Second-Generation Cryoballoon Ablation

**DOI:** 10.6061/clinics/2020/e1672

**Published:** 2020-07-06

**Authors:** Chen-Feng Zhang, Jing-Lan Wu, Ling You, Ying Yang, Bo-Fei Ma, Rui-Qin Xie

**Affiliations:** Department of Cardiovascular Medicine, The Second Hospital of Hebei Medical University, Shijiazhuang, Hebei, China

**Keywords:** Atrial Fibrillation, Cryoballoon Ablation, Pulmonary Vein Isolation, Thawing Rate

## Abstract

**OBJECTIVE:**

To evaluate whether thawing rate could be a novel predictor of acute pulmonary vein isolation (PVI) and explore the predictive value of thawing rate as a factor ensuring long-term PVI (vagus reflex).

**METHODS:**

A total of 151 patients who underwent cryoballoon ablation for atrial fibrillation (AF) were enrolled in this retrospective study between January 2017 and June 2018. The thawing rate was calculated using the thawing phase of the cryoablation curve. Receiver operating characteristic (ROC) curve was used to analyze the predictive value of the thawing rate for acute PVI and vagus reflex.

**RESULTS:**

ROC curve analyses revealed that the interval thawing rate at 15°C (ITR15) was the most valuable predictor of PVI, with the highest area under curve (AUC) value of the ROC curve. The best cut-off value of ITR15 for PVI was ≤2.14°C/S and its sensitivity and specificity were 88.62% and 67.18%, respectively. In addition, the ITR15 of the successful PVI group after cryoballoon ablation was significantly slower than the failed PVI group. ITR15 was a predictor of vagus reflex and the occurrence of vagus reflex group had a slower ITR15 compared to the non-occurrence group.

**CONCLUSIONS:**

Thawing rate was a novel predictor of acute PVI and the ITR15 was the most valuable predictor of acute PVI. In addition, ITR15 was a predictive factor ensuring long-term PVI (vagus reflex). Our study showed that thawing rate may serve in the early identification of useless cryoballoon ablation.

## INTRODUCTION

Pulmonary vein isolation (PVI) is the main treatment for atrial fibrillation (AF) (1). Cryoballoon ablation as an efficient and relatively safe procedure that is increasingly being used to perform PVI in patients with AF in recent years (2,3). Cryoballoon ablation is not inferior to radiofrequency ablation with respect to efficacy for the treatment of AF and there is a lower re-hospitalization rate and re-ablation rate in cryoablation ablation (4,5). Previous studies have shown that the second-generation cryoballoon is an effective and safe technique in achieving both favorable clinical outcomes and acute PVI ([Bibr B06] [Bibr B07] [Bibr B08] 6-9). However, recently published data indicates that approximately 90% of PVs are still isolated 3 months after second-generation cryoballoon ablation (10). Despite encouraging results, recurrence of AF is still relatively frequent after surgery, which may be related to PV reconnection ([Bibr B10] [Bibr B11] 10-12). To date, limited information is available regarding the factors determining PV reconnection following cryoballoon ablation.

Previous studies have demonstrated that cryoablation temperature, cryoballoon freeze duration, cryoablation duration, achievement of −40°C within 60s, and balloon warming time are predictors of late PV reconnections after cryoballoon ablation for the treatment of AF ([Bibr B13] [Bibr B14] [Bibr B15] 13-16). However, the thawing rate, which is related to time and temperature, lacks further research. At the same time, basic experiments have proved that freezing and thawing rates are related to freezing injury, which can cause PVI; therefore, it has practical research significance and clinical value (17,18). In addition, previous studies have proved that vagus reflex could independently predict long-term PVI (19). However, there was yet no systematic study based on thawing rate and factors ensuring long-term PVI. In this study, we aimed to evaluate whether thawing rate could be a novel predictor of acute PVI and explore the predictive value of the thawing rate for vagus reflex.

## MATERIALS AND METHODS

### Patients

We retrospectively reviewed 151 patients (21 cases of persistent AF and 130 cases of paroxysmal AF) who received cryoballoon ablation for AF using the single big (28 mm) second-generation cryoballoon technique from January 2017 to June 2018. The inclusion criteria were as follows: 1) patients with symptomatic AF who were not responding to class I and III antiarrhythmic drugs; 2) patients with age <80 years; 3) patients who underwent cryoballoon ablation for AF using the single big (28 mm) second-generation cryoballoon technique. The exclusion criteria were as follows: 1) history of atrial fibrillation ablation; 2) presence of atrial thrombosis; 3) presence of valvular disease (moderate and severe valvular stenosis, severe valvular regurgitation); 4) history of prosthetic heart valve replacement; 5) pregnant women; 6) patients with severe liver and kidney dysfunction; 7) patients with malignant tumors or hematological diseases. This study was approved by the ethics committee of the Second Hospital of Hebei Medical University. Written informed consent was obtained from each patient.

### Cryoballoon ablation procedure

Cryoballoon ablation was carried out by the conventional method, as described previously (20). A 3-dimensional computed tomography (Aquilion ONETM, TSX-301C, Toshiba Medical Systems, Tokyo, Japan) was performed in the week prior to the procedure. Patients were placed under general anesthesia. The goal of the cryoablation was electrical PV isolation of the four major PVs (left inferior pulmonary vein [LIPV], left superior pulmonary vein [LSPV], right inferior pulmonary vein [RIPV], right superior pulmonary vein [RSPV]) as confirmed by entrance and/or exit blocks. A transseptal puncture was performed using an RF needle (Baylis Medical, Inc., Montreal, QC, Canada). After passing through the transseptal sheath, the cryoballoon catheter entered into the left atrium and it was continuously infused with heparin saline through a 12F oriented sheath. Following all PV venography during raid ventricular pacing, the second-generation cryoballoon system (Arctic Front Advance™ Cardiac Cryoablation Catheter, Medtronic, Minneapolis, MN) was advanced and placed on the ostia of each PV using an inner circular mapping catheter (Achieve, Medtronic). The size of the balloon was selected as 28 mm according to the measurement of the PV ostia from the venograms and computed tomographic images. The cryoballoon was delivered and moved along the Achieve catheter until the pulmonary vein ostia was completely blocked (blocking was determined by pulmonary venography).

In general, each pulmonary vein was cryoablated at least twice for 180 seconds each time. After cryoablation, the Achieve catheter was used to test whether the PVI was completed. If two cryoablations failed to complete the PVI, cryoablation would continue until PVI was successful. In the implementation of right PVI, a secondary catheter was required to be placed at the phrenic potential of the superior vena cava. A cycle length of 999 ms phrenic nerve pacing was taken to continuously monitor the right phrenic nerve function. If the balloon nadir temperatures reached a temperature below -55°C or if the diaphragm movement were weakened or disappeared, the cryoablation would be immediately stopped. A total of 604 cryoablations were performed. Twelve of the cryoablations were excluded, including five cases of hypothermia, six cases of inability to determine isolation, and one case of sacral nerve movement disappearance. The remaining 592 cryoablations were analyzed.

### Cryoablation curve

**Figure 1:**
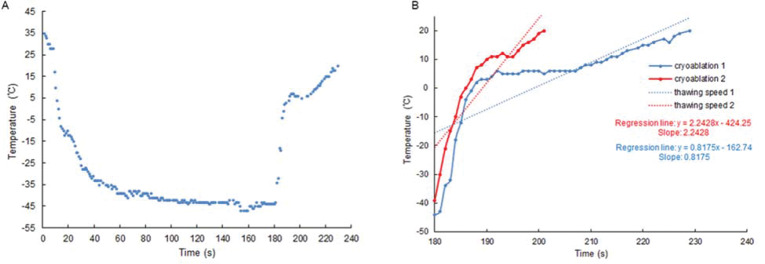
Examples of cryoablation curves and their fitted regression line A, Example of a complete cryoablation curve. B, Examples of a portion of the cryoablation curve (the time frame from the first value >-50°C, with 60s duration) and their fitted regression line.

To avoid the cumulative effect of ablation, we only analyzed the first ablation of each pulmonary vein. The Cryoconsole software (Medtronic CryoCathLP) recorded the cryoablation curves in detail and we only analyzed the thawing phases of the cryoablation curves. The thawing phase was from the end of freezing to the end of thawing, in which the thawing rate was generated. Cryoablation curves from 592 cryoablations were reconstructed using these data (Figure 1A). Our aim was to quantify the thawing rate by fitting a regression line to a specific portion of the cryoablation curve (21). The slope of the regression line (Figure 1B) provided an immediate measure of the rapidity of the temperature rise. Furthermore, this method had the advantage of averaging the information of several samples, thus compensating for a certain amount of imprecision.

### Statistical analysis

All statistical analyses were performed by using SPSS version 22.0 (SPSS Institute. IL.USA). Quantitative data were expressed as means±standard deviations (SD) and were compared using Student’s t-test. Qualitative data were expressed as number and percentage. Diagnostic performance of the thawing rate as a predictor of PVI was assessed by constructing a receiver operating characteristic (ROC) curve. By calculating the area under curve (AUC) of different thawing rates, the thawing rate interval with the greatest predictive value for acute PVI was obtained. Vagus reflex was defined as sinus bradycardia (<40 bpm), asystole, atrioventricular (AV) block, or hypotension that occurred within cryoapplication (22). ROC curve was used to analyze the predictive value of the thawing rate for vagus reflex. Statistical significance was set at *p*<0.05.

## RESULTS

### Baseline characteristics

A total of 151 patients (81 males, 70 females; mean age: 61.32±10.22 years; 21 cases of persistent AF and 130 cases of paroxysmal AF) who received cryoballoon ablation for AF were enrolled in this retrospective study from January 2017 to June 2018. The baseline characteristics of participants are showed in Table 1. Among the 151 patients, 80 (52.98%) patients had hypertension and 26 (17.22%) had diabetes. Otherwise, 23 (15.23%) patients had a history of smoking, 19 (12.58%) patients had a history of drinking, and 34 (22.52%) patients had coronary heart disease.

### Thawing rate predicts acute PVI

**Figure 2:**
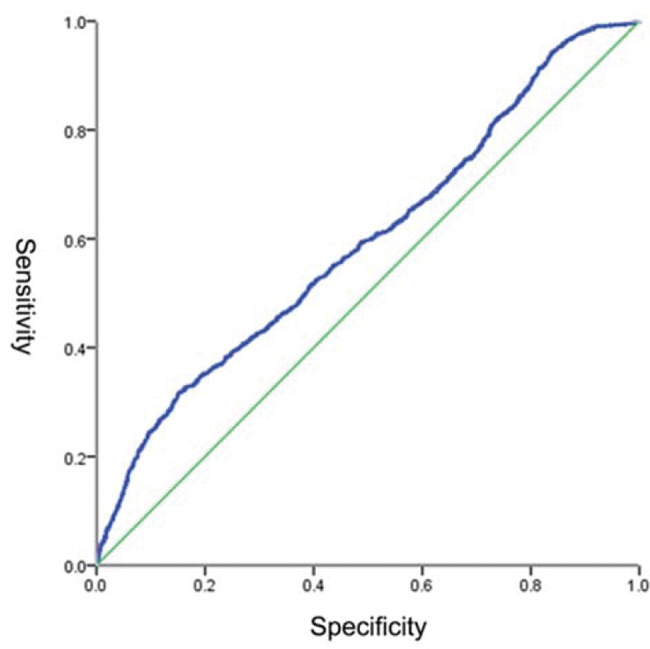
ROC curve analysis. ROC, receiver operating characteristic.

We first evaluated whether thawing rate could be a novel predictor for acute PVI. The ROC curve analysis results showed that thawing rate was a predictor (AUC=0.596) of acute PVI. The best cut-off value of thawing rate for acute PVI was≤2.21°C/S and its sensitivity and specificity were 31.76% and 84.85%, respectively (Figure 2).

### ITR15 as the most valuable predictor of acute PVI

**Figure 3:**
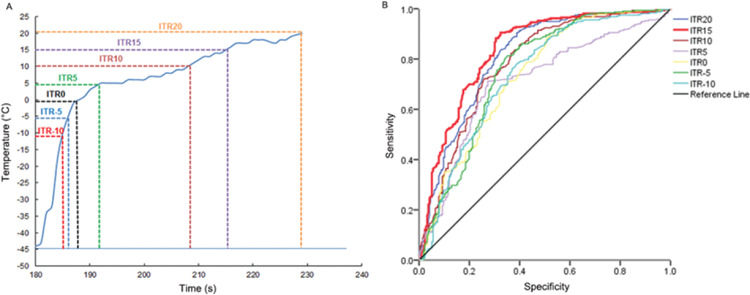
ROC curve of different thawing rate intervals. A, Thawing curves were divided into seven intervals. ITR-10, interval thawing rate at -10°C; ITR-5, interval thawing rate at -5°C; ITR0, interval thawing rate at 0°C; ITR5, interval thawing rate at 5°C; ITR10, interval thawing rate at 10°C; ITR15, interval thawing rate at 15°C; ITR20, interval thawing rate at 20°C. B, ROC curve of different thawing rate intervals. ROC, receiver operating characteristic.

In order to obtain the thawing rate interval with the greatest predictive value for acute PVI, we selected the interval from the start of thawing to -10°C, -5°C, 0°C, 5°C, 10°C, 15°C, 20°C (Figure 3A). By calculating the AUC of different thawing rate, it was found that the maximum AUC was for interval thawing rate at 15°C (ITR15) (Figure 3B, Table 2). It showed that the ITR15 (thawing rate≤2.14°C/s; AUC, 0.823; 95% CI, 0.788-0.859) was the most valuable predictor of PVI. ITR15 presented 88.62% sensitivity and 67.18% specificity, with a 77.2% of positive predictive value (PPV) and 82.5% negative predictive value (NPV).

**Figure 4:**
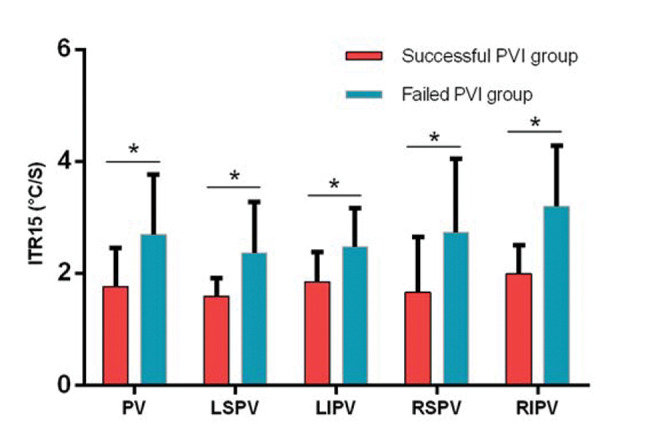
ITR15 of different PVs during cryoballoon ablation PV, pulmonary vein; LSPV, left superior pulmonary vein; LIPV, left inferior pulmonary vein; RSPV, right superior pulmonary vein; RIPV, right inferior pulmonary vein; PVI, pulmonary vein isolation; ITR15, interval thawing rate at 15°C; *p<0.05.

To further verify our results, we compared the ITR15 of the left superior, left inferior, right superior, right inferior pulmonary veins and total pulmonary veins in the successful PVI group and the failed PVI group, respectively. The results showed that the ITR15 of the successful PVI group after cryoballoon ablation was significantly slower than that of the failed PVI group (*p*<0.05) in different PV (Figure 4).

### ITR15 predict vagus reflex

**Figure 5:**
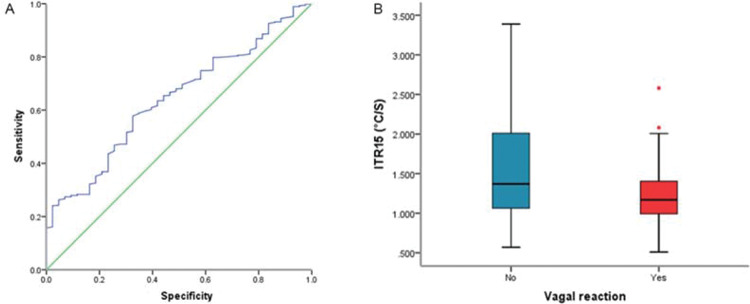
Thawing rate and vagus reflex A, ROC curve analysis. B, Comparing ITR15 of the occurrence of vagus reflex group and the non-occurrence group. ITR15, interval thawing rate at 15°C; ROC, receiver operating characteristic.

A total of 43 vagal reflexes were observed during cryoablation. Most vagus reflexes (93.02%) occurred in thawing phase and within 1-3 minutes after the end of thawing. The highest occurrence of vagus reflex was in LSPV (60.47%) and the vagus reflexes occurred in LIPV, RSPV, and RIPV occupying 32.56%, 2.32% and 4.65%, respectively. The ROC curve analysis results showed that ITR15 was a predictor (AUC, 0.64; best cut-off point≤1.79°C/s; sensitivity, 67.78%) of vagus reflex (Figure 5A). Meanwhile, the occurrence of vagus reflex group had a slower ITR15 compared to the non-occurrence group (1.77±0.47°C/S *vs* 2.22±1.01°C/s, *p*<0.01) (Figure 5B).

## DISCUSSION

Cryoballoon ablation is increasingly being used to perform PVI in patients with AF (23). However, there is still a lack of effective indicators to assess the effectiveness of cryoballoon ablation. Previous studies had shown that cryoablation temperature and cryoablation duration could predict acute PVI, but the time and temperature were natural and not artificial results in the process of cryoablation (13,15). Therefore, although sometimes the cryoablation temperature and time could not meet the expectation of the operator, PVI had already been completed, which made the operator unable to judge the results, leading to excessive increase in the number and time of cryoablation. However, little research has been done with regard to the thawing rate, which was related to time and temperature. In this study, we found that thawing rate was a novel predictor of acute PVI, which could provide a certain reference value when factors such as cryoablation temperature and time cannot effectively predict PVI. Deubner et al. found that a 20-second time frame with a freezing rate of <17°C could best predict PVI (21). Meanwhile, our study showed that the ITR15 was the most valuable predictor of acute PVI.

How could freezing and thawing rates predict PVI? From a cellular perspective, experimental studies had demonstrated that freezing and thawing rates could affect cell death. The faster the freezing rate, the more cell death could occur. With rapid cooling, the cell fluid had not yet been removed from the cells under the action of osmotic pressure, which aggravated the degree of intracellular freezing, leading to excessive cell death (17,18,24). The slower the thawing rate, the more cell death could occur. Slow thawing may promote the entry of extracellular fluid into cells, resulting in cell and tissue damage (25). Overall, thawing and freezing rates could cause myocardial cell damage in the PV or vestibule of the PV, which was essential for PVI. From the cryoballoon ablation application perspective, rapid freezing and thawing rates were predictors of better PV occlusion; therefore, there were better circumferential contact area and better cryoablation effects. The application of complete occlusive cryoballoon ablation could shorten the contact distance between the balloon and the frozen surface, resulting in a lower temperature on the contact surface, which was essential for long-term PVI.

A previous study had proved that increased vagal reflex could reduce the recurrence rate of AF (19). Our study showed that ITR15 was a predictor of vagus reflex, although it had a lower predictive value (AUC=0.64), which probably due to vagal reflex may be more closely related to the distribution of vagus nerve at the junction of the atrium and PV (19). However, the occurrence of vagus reflex group had a slower thawing rate compared to the non-occurrence group. That means, even if this ablation occurred on the vagus nerve, vagus reflex would not occur without a slow thawing rate. In summary, thawing rate was associated with the prognostic factor that may affect long-term PVI.

There were several limitations in the current study. First, this study was a retrospective study conducted at a single center, which had certain limitations in clinical application. Second, the number of patients included in this study was small. Third, the patients with recurrent AF were less likely to opt for a second surgery because of different state systems, different national conditions and other factors. We were not sure which PV was reconnected; therefore, we were not sure if the thawing rate really had a predictive value for long-term PVI. Fourth, we used a single big (28 mm) second-generation cryoballoon technique. Due to the fact that temperature characteristics may vary with balloon size, our analysis only applied to the 28-mm balloon. Fifth, the complex anatomical structure and size of the pulmonary vein were not considered, which may affect the ablation effect. Lastly, we did not compare the thawing time of the ITR15 in the successful and failed PVI groups with the reported predictors.

## CONCLUSION

Thawing rate was a novel predictor of acute PVI and the ITR15 was the most valuable predictor of acute PVI. In addition, ITR15 was a predictive factor ensuring long-term PVI (vagus reflex). Our study showed that thawing rate may serve in the early identification of useless cryoballoon ablation.

## AUTHOR CONTRIBUTIONS

Zhang CF and Xie RQ were responsible for the study conception and design, and manuscript critically review. Zhang CF and Yang Y were responsible for the data collection. Zhang CF, Wu JL and You L were responsible for the data analysis and interpretation. Zhang CF was responsible for the manuscript drafting article. Zhang CF, Ma BF and Wu JL were responsible for the statistics. Xie RQ was responsible for the administrative support. All of the authors have read and approved the final version of the manuscript.

## Figures and Tables

**Table 1:** Baseline characteristics of the included patients.

Characteristics	Number (n,%)
Age (years), Mean±SD	61.32±10.22
Sex(n)	
Male	81 (53.64)
Female	70 (46.36)
Paroxysmal AF	130 (86.09)
Smoking history	23 (15.23)
Drinking history	19 (12.58)
Hypertension	80 (52.98)
Diabetes	26 (17.22)
Coronary heart disease	34 (22.52)
CHA2DS2-Vasc	2.04±1.56
Ultrasound parameters	
LAAEV (cm/s)	62.55±23.16
LAD (mm)	35.65±3.78
LVEF (%)	61.19±7.93
Pulmonary vein diameter (cm)	1.66±0.37
Procedural variables	
Procedure time (min)	66.68±36.03
Fluoroscopic time (min)	10.64±5.85
Cryoablation variables	
Nadir temperature (°C)	-44.12±7.94
Freeze-time (s)	2.21±1.01
Thaw time (s)	39.97±16.62

AF, atrial fibrillation; LAAEV, emptying velocity of left atrial appendage; LAD, left atrial diameter; LVEF, left ventricular ejection fraction.

**Table 2:** AUC of different thawing rate intervals.

Interval	AUC	95% CI	*p*-value
ITR-10	0.736	0.692-0.781	<0.01
ITR-5	0.754	0.710-0.799	<0.01
ITR0	0.733	0.689-0.778	<0.01
ITR5	0.709	0.663-0.754	<0.01
ITR10	0.783	0.742-0.824	<0.01
ITR15	0.823	0.788-0.859	<0.01
ITR20	0.81	0.772-0.849	<0.01

AUC, area under curve; 95% CI, 95% confidence interval; lITR-10, interval thawing rate at -10°C; ITR-5, interval thawing rate at -5°C; ITR0, interval thawing rate at 0°C; ITR5, interval thawing rate at 5°C; ITR10, interval thawing rate at 10°C; ITR15, interval thawing rate at 15°C; ITR20, interval thawing rate at 20°C.
